# Low hybridization temperatures improve target capture success of invertebrate loci: a case study of leaf-footed bugs (Hemiptera: Coreoidea)

**DOI:** 10.1098/rsos.230307

**Published:** 2023-06-28

**Authors:** Michael Forthman, Eric R. L. Gordon, Rebecca T. Kimball

**Affiliations:** ^1^ California State Collection of Arthropods, Plant Pest Diagnostics Branch, California Department of Food and Agriculture, 3294 Meadowview Road, Sacramento, CA 95832, USA; ^2^ Entomology and Nematology Department, University of Florida, 1881 Natural Area Drive, Gainesville, FL 32611, USA; ^3^ Department of Ecology and Evolutionary Biology, University of Connecticut, 75N. Eagleville Road, Unit 3043, Storrs, CT 06269, USA; ^4^ Department of Biology, University of Florida, 876 Newell Drive, Gainesville, FL 32611, USA

**Keywords:** annealing temperature, exons, Pentatomomorpha, touchdown hybridization, ultraconserved elements

## Abstract

Target capture is widely used in phylogenomic, ecological and functional genomic studies. Bait sets that allow capture from a diversity of species can be advantageous, but high-sequence divergence from baits can limit yields. Currently, only four experimental comparisons of a critical target capture parameter, hybridization temperature, have been published. These have been in vertebrates, where bait divergences are typically low, and none include invertebrates where bait-target divergences may be higher. Most invertebrate capture studies use a fixed, high hybridization temperature to maximize the proportion of on-target data, but many report low locus recovery. Using leaf-footed bugs (Hemiptera: Coreoidea), we investigate the effect of hybridization temperature on capture success of ultraconserved elements targeted by (i) baits developed from divergent hemipteran genomes and (ii) baits developed from less divergent coreoid transcriptomes. Lower temperatures generally resulted in more contigs and improved recovery of targets despite a lower proportion of on-target reads, lower read depth and more putative paralogues. Hybridization temperatures had less of an effect when using transcriptome-derived baits, which is probably due to lower bait-target divergences and greater bait tiling density. Thus, accommodating low hybridization temperatures during target capture can provide a cost-effective, widely applicable solution to improve invertebrate locus recovery.

## 1. Introduction

Many biological disciplines have witnessed a dramatic increase in the amount of genomic data sampled due to recent advances and declining cost of next-generation sequencing technologies. While low-coverage, whole-genome sequencing may be cost-effective for some research questions, genome reduction approaches that enrich for genomic regions of interest prior to high-throughput sequencing are often more feasible (reviewed in [1]). Some of the most frequently employed genome reduction techniques in phylogenomic, ecological and functional genomic studies are target capture approaches, which include exon capture, anchored hybrid enrichment (AHE) and capture of ultraconserved elements (UCEs) (e.g. [2–5]). In general, target capture leverages existing genomic resources to synthesize short (60–120 bp) nucleotide bait sequences complementary to genomic regions of interest. Baits are then hybridized to DNA libraries, and unbound DNA (i.e. non- or off-targets) is removed via a series of washing steps prior to sequencing.

Target capture approaches have been widely applied to a diversity of taxa at various evolutionary timescales, e.g. birds [3], lizards [6], fish [7], plants [8], spiders [9], amphibians [10] and insects [11]. While vertebrate capture studies often recover a relatively high proportion (greater than 50%) of targeted loci (e.g. [3,5,12]), many invertebrate studies suffer from a much lower yield, particularly when highly conserved loci like UCEs are targeted (e.g. [9,11,13–16]). While these and other studies may vary in the conditions of the capture experiment, the often-consistent disparity in the proportion of loci recovered between vertebrate and invertebrate target capture experiments suggests that capture dynamics may differ due to greater bait-target divergences among invertebrate taxa compared with many vertebrates [17–19].

Optimizing existing invertebrate target capture bait sets to be more tailored to focal taxa has been shown to improve recovery (e.g. [20,21]), suggesting that baits may often be too divergent from some taxa to allow effective recovery. However, genomic resources that permit such optimization for many other groups are still lacking. As such, optimizing one or more *in vitro* target capture conditions may provide a more cost-effective solution to improve locus recovery. Recent studies in various non-invertebrate taxa have investigated several parameters that may affect the success of *in vitro* target capture approaches, including, e.g. GC content and tiling of baits, starting amount of DNA or baits, bait-target divergence and washing stringency (e.g. [5,22,23]). Another condition that can affect capture success is hybridization temperature during bait-target annealing (table 1).

**Table 1.** Experimental design of four studies that investigated the effects of target capture hybridization temperatures on various metrics used to assess capture performance.

characteristics of study	Li *et al*. [5]	Paijmans *et al*. [24]	Cruz-Dávalos *et al*. [23]	Mohandesan *et al*. [25]
taxon	Gnathostomata	Felidae	Equidae	*Camelus* (Camelidae)
target data type	CDS	mitogenome	SNP	mitogenome
no. of targets	1449	1 (entire mitogenome)	approximately 5000 (SNPs)	1 (entire mitogenome)
bait type	biotinylated RNA	‘oligonucleotides’	biotinylated RNA	biotinylated RNA
bait length (bp)	120	60	60	80
tiling density	2x	30x	3x	4x
sample quality	fresh	fresh and degraded	degraded	degraded
target capture method	in-solution	solid-state	in-solution	in-solution
capture protocol (CP) 1	65°C hybridization (duration not reported)	65°C hybridization; captured targets used for additional capture under same conditions (duration not reported)	65°C hybridization (24 h)	65°C hybridization (36 h)
capture protocol (CP) 2	65°C to 50°C hybridization (5°C decrease every 11 h)	same as CP1 but 50°C hybridization	55°C hybridization (40 h)	65°C to 50°C hybridization (5°C decrease every 12 h)
capture protocol (CP) 3	CP3 used captured targets and same capture conditions from CP 2	65°C to 50°C hybridization (5°C decrease every 16.25 h); captured targets used for additional capture under same conditions		
metrics evaluated	proportion of target loci recovered; GC content; bait-target divergence; target length recovered; chromosomal position	proportion of on-target reads; target coverage; bait-target divergence (fresh tissues only)	proportion of on-target reads; target coverage; fold enrichment; read length	proportion of on-target reads; percentage of endogenous DNA; percentage of PCR duplicate reads
recommended CP based on metrics	CP 3	CP 3 (fresh); CP 1 (archival and ancient)	CP 2 (samples with low to medium endogenous DNA); CP 1 and CP 2 not significantly different (samples with high endogenous DNA)	CP 1 and CP 2 performance not significantly different
relevant conclusions	lower hybridization temperatures improve proportion of target loci recovered, particularly when bait-target divergence is high	sample type and quality probably determines which hybridization temperature results in best capture based on proportion of on-target reads; higher temperatures may be better for degraded samples with greater amounts of contamination	lower hybridization temperatures result in greater enrichment when samples have greater amounts of contamination but no impact when samples have higher amounts of endogenous DNA	lower hybridization temperatures perform similarly with standard capture conditions for recovery of uniquely mapped reads or capture efficiency

Invertebrate target capture studies typically employ a fixed hybridization temperature at 65°C as suggested by standard protocols (we note that temperatures are often not reported in AHE studies) (e.g. [11,13,14,16]). However, lower hybridization temperatures, whether fixed or achieved through touchdown (i.e. incremental decreases in temperature during target capture), may improve on-target and locus recovery due to relaxed specificity and increased sensitivity between baits and targets (e.g. [5,24]). This may be particularly advantageous if some baits are more divergent from their targets (as is commonly the case in invertebrates) or have lower optimal annealing temperatures than other baits [5,26]. However, relaxing specificity to allow for partial matching between baits and targets should also increase the risk of baits to potentially hybridize with paralogous sequences exhibiting some degree of divergence from the corresponding target sequence and/or may increase the number of off-target sequences (e.g. [23]) and thus reduce read numbers for targeted regions. While a few invertebrate capture studies have used lower temperatures during bait-target hybridization [27–32], no studies have experimentally investigated the effect of altering hybridization temperatures on capture success of targeted loci in invertebrates.

Here, we evaluated the impact of four different protocols that varied hybridization temperatures on invertebrate target capture success in leaf-footed bugs and allies (Hemiptera: Coreoidea) and closely related taxa using samples from various sources (fresh versus older, dried specimens) and library qualities to reflect conditions typical of empirical studies. One protocol used a fixed, standard hybridization temperature (65°C) while the remaining three protocols employed touchdown approaches with different final temperatures ranging as low as 50°C. While lower temperatures could have been investigated, we selected 50°C as the lowest temperature, given this has been commonly used in comparisons of hybridization temperatures in target capture experiments (table 1). Specifically, we addressed the following questions: (i) do touchdown target capture approaches with lower hybridization temperatures result in a greater total of on-target reads, total assembled contigs, total targeted loci and longer targeted assemblies compared with the commonly used standard hybridization temperature?; (ii) does the touchdown target capture protocol with the lowest final temperature (i.e. 50°C) produce the most data for the variables listed in question 1 or is there no further improvement or benefit beyond intermediate temperatures (i.e. between 65°C and 50°C)? and (iii) does a lower hybridization temperature from touchdown capture protocols generate a greater proportion of off-target reads and/or paralogous sequences?

Our study used a subsampled version of an existing Hemiptera-derived UCE bait set [33,34], but we also introduced newly designed baits (with slightly greater tiling) derived from coreoid transcriptomes and evaluated the ability of these new baits to enrich samples *in vitro*. Given the different bait designs, we also examined whether the effects of hybridization temperature exhibited different patterns across bait design strategies; specifically, we investigated if (i) the proportion of on-target reads and read depth exhibit similar trends across hybridization temperature conditions regardless of bait design strategy; (ii) the capture of loci with greater divergences from baits shows the greatest improvement at lower hybridization temperatures than loci with less divergence from their baits; (iii) the number of putative paralogous loci recovered increases as hybridization temperature decreases regardless of bait design strategy; and (iv) an increase in bait tiling improves read depth of captured loci.

### 1.1. Novelty of the present study

Based on recent reviews (e.g. [35]) and our own search of prior literature, only four studies—all in vertebrates—have explicitly tested the impacts of lower hybridization temperatures by comparing two temperature conditions (table 1). These studies have provided *mixed* results and recommendations on the *benefits* of implementing lower temperatures in capture experiments based on various metrics (table 1). Thus, additional studies comparing different hybridization temperatures are needed. This is particularly true for systems in which bait-target divergences are much higher than many of the studies in table 1 and for which prior target capture studies have recovered a low proportion of targeted loci.

Our study addresses this gap by studying a group of invertebrates that are approximately 160–230 Myr old [36–38] and for which target capture studies have yielded low numbers of recovered loci (e.g. less than 40% [34]), which is probably due to high bait-target divergences. Our study also explores the effects of hybridization temperatures on other aspects of capture success not previously investigated in the studies listed in table 1, such as the number of putative paralogues recovered between different hybridization temperature conditions. Lastly, we explore intermediate temperatures to evaluate whether there is no further improvement to locus recovery or if costs outweigh the benefits at a particular temperature, which has also not been investigated by the studies in table 1.

## 2. Materials and methods

### 2.1. Sample material

**Figure 1.**
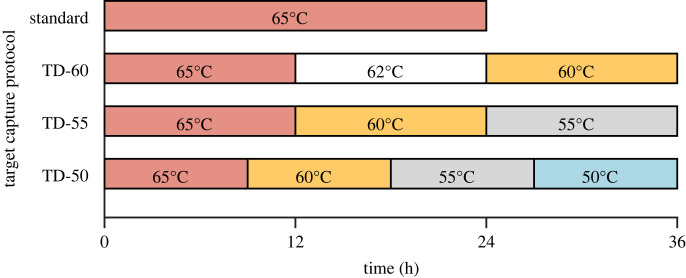
Experimental target capture set-up. Hybridization temperatures across time (in hours) are reported. Abbreviations: TD-60, touchdown hybridization approach starting at 65°C for 12 h followed by 62°C for 12 h and ending at 60°C for 12 h; TD-55, touchdown hybridization approach starting at 65°C for 12 h followed by 60°C for 12 h and ending at 55°C for 12 h; TD-50, touchdown hybridization approach starting at 65°C for 9 h followed by 60°C for 9 h, 55°C for 9 h and ending at 50°C for 9 h.

Our target capture experiment was performed on 39 taxa (36 species of Coreoidea, three outgroup taxa), of which 30 were ethanol (EtOH), frozen or silica bead (fresh) preserved samples (collected 2008–2017) and nine were degraded samples from pinned museum material of varying ages (1935–2017) (electronic supplementary material, table S1). These 39 taxa were selected for this experiment based on the availability of extra DNA libraries for additional target captures and to include a diversity of preservation methods and specimen ages (recent/fresh versus historical/dried), as well as library qualities (best, moderate and marginal quality based on initial sequencing outcomes relative to other samples). All taxa had previously been subjected to target capture protocols and sequenced prior to the start of this study (i.e. freshly preserved samples or dried preserved samples were subjected to the standard or TD-60 protocols shown in figure 1, respectively); capture data for 27 taxa have already been published following protocols described in [29,30,34] (see electronic supplementary material, table S1 and references therein).

### 2.2. Target capture baits

**Figure 2.**
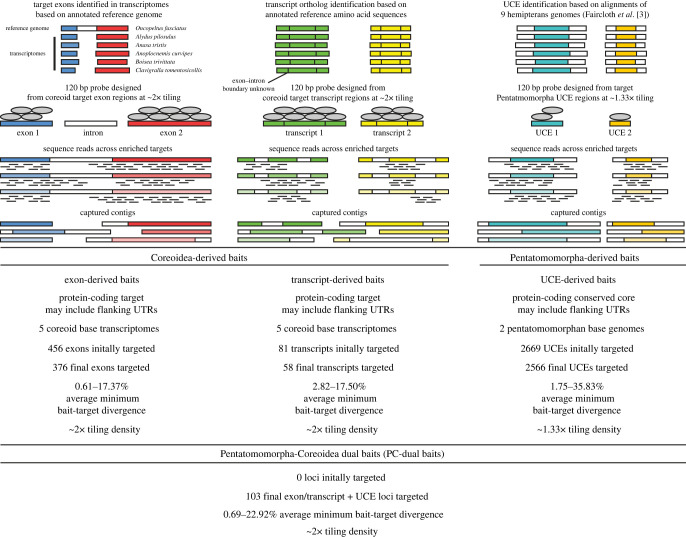
Design and properties of baits used in this study. Abbreviations: bp, base pair; UCE, ultraconserved element; UTR, untranslated region.

For a list of terms and their definitions used in this study see table 2. A summary of bait properties from our different bait design strategies are given in figure 2 and are briefly discussed below.

**Table 2.** Terminology related to bait design and targeted loci.

term	definition
ultraconserved element (UCE) baits	120 bp baits designed to target highly conserved genomic regions at approximately 1.33x tiling. A Hemiptera-wide UCE bait set was designed by [33], which targets the conserved core of protein-coding loci [16]; however, contigs captured by UCE baits may also include introns and/or untranslated regions (UTRs).
Pentatomomorpha-derived baits	The Hemiptera-wide UCE bait set [33] was subsampled to include only those baits designed from the genomes of two species within the infraorder Pentatomomorpha, which are the closest relatives to the ingroup of this study.
exon-derived baits	120 bp baits designed at approximately 2x tiling from individual exon sequences (i.e. baits do not span across introns) exhibiting greater than or equal to 60% conservation across transcriptomes from five species of Coreoidea. While the baits do not target UTRs, given that they are derived from transcriptome sequences, contigs captured by these baits may include introns and/or UTRs.
transcript-derived baits	120 bp baits designed at approximately 2x tiling from sequences that may include one or more exons and that exhibit greater than or equal to 60% conservation across transcriptomes from five species of Coreoidea. While the baits do not target UTRs, given that they are derived from transcriptome sequences, baits may span across one or more introns, and thus contigs captured by these baits may include introns and/or UTRs.
Coreoidea-derived baits	Baits designed primarily from transcriptomes of five species of Coreoidea (i.e. exon- and transcript-derived baits combined).
Pentatomomorpha-Coreoidea (PC) dual baits	Loci targeted by Pentatomomorpha-derived UCE baits as well as by exon-derived or transcript-derived baits (i.e. Coreoidea-derived baits).

We used a previously published custom myBaits kit [34], which subsampled a Hemiptera-wide derived UCE bait set (at approximately 1.33x tiling) designed by [33] to only include two pentatomomorphan taxa that are more closely related to but not included in our ingroup taxa (herein, referred to as ‘Pentatomomorpha-derived baits'; table 2; figure 2). We also introduce an independently designed set of baits (120 bp; approximately 2x tiling) derived from five coreoid transcriptomes [39,40] using two strategies: baits were designed from individual exons (herein, ‘exon-derived baits’) while others were designed across entire transcripts (herein, ‘transcript-derived baits'; baits from both strategies collectively referred to as ‘Coreoidea-derived baits’; table 2; figure 2). Specific details on bait design procedures for the Coreoidea-derived baits are in the electronic supplementary material.

### 2.3. DNA extraction, library preparation and target capture

See references in the electronic supplementary material, table S1 for details on library preparation and target capture of previously published samples. For new samples, we extracted genomic DNA from any part of the body or the entire body from EtOH-preserved, silica bead preserved, frozen or dried specimens to sample similar amounts of tissue across taxa, where possible (electronic supplementary material, table S1), and constructed libraries following procedures we have used elsewhere (e.g. [30,31,34]) and outlined in the electronic supplementary material, methods.

To evaluate the effects of different hybridization conditions on target capture, we compared four experimental protocols using 1/2 or 1/4 volume baits for fresh and dried samples, respectively, which differed only in hybridization temperatures used over a 24 (common in standard target capture protocols) or 36 h period (figure 1). Due to the limited availability of extra DNA libraries, previously sequenced samples could only be assigned to one of three different target capture protocols (see electronic supplementary material, table S2). In assigning samples to protocols, we attempted to distribute sample preservation methods, sample age and library quality (based on our initial sequencing efforts).

All post-capture protocols followed [34], with the exception that captures were washed at temperatures corresponding to the final hybridization temperature used in each capture protocol (i.e. 65°C for standard, 60°C for TD-60, etc.). All enriched library pools were combined in equimolar amounts and sequenced on an Illumina HiSeq3000 lane (2 × 100) at the University of Florida's Interdisciplinary Center for Biotechnology Research.

### 2.4. Sequence data processing and analysis

Unless otherwise stated, all data processing steps and analyses mentioned below used default settings. Sequence reads were demultiplexed by the sequencing facility. Adapters were trimmed with illumiprocessor [41,42]. Duplicate reads were filtered with PRINSEQ-lite. Reads were then error corrected using QuorUM and subsequently assembled de novo using SPAdes v. 3.13.0 with the single-cell and auto coverage cut-off options invoked [43–45]. PHYLUCE v. 1.5.0 [46] was then used to extract targeted loci from assembled contigs.

Because our preliminary comparison of loci targeted by Coreoidea-derived baits against the Pentatomomorpha-derived baits prior to bait design indicated that some loci were targeted by both sets of baits (see electronic supplementary material for details), a more thorough confirmation was performed after *in vitro* target capture. Using captured loci targeted by our Coreoidea-derived baits, we performed a tblastx (e-value threshold = 1 × 10^−10^) search against those loci captured by the Pentatomomorpha-derived baits and extracted matches with ALiBaSeq [47]. Of the loci captured by the Pentatomomorpha-derived baits, we found that 103 of these were targeted by both Pentatomomorpha- and Coreoidea-derived baits. It is worth noting that during this process, we also determined that some targeted transcript loci were also targeted by multiple, adjacent UCE loci by Pentatomomorpha-derived baits; in such cases, we treated these loci as a single locus. Thus, we had 376 loci targeted by exon-derived baits, 58 targeted by transcript-derived baits, 2566 targeted by Pentatomomorpha-derived baits and 103 targeted by both Pentatomomorpha- and Coreoidea-derived baits (herein, ‘Pentatomomorpha-Coreoidea (PC) dual baits'; table 2; figure 2), resulting in a total of 3103 targeted loci.

We calculated the number and lengths of assembled contigs and captured loci using PHYLUCE. Because our Pentatomomorpha- and Coreoidea-derived baits were designed from different sets of genomes and transcriptomes of varying divergences, we also calculated the average minimum distances between our baits and captured loci for exon-derived, transcript-derived, Pentatomomorpha-derived and PC dual baits. We then calculated read depth using our filtered reads and the total number of on-target filtered reads using BBMap v. 38.44 [48]. We determined the number of captured loci with putative paralogues by invoking the keep-duplicates option in the PHYLUCE phyluce_assembly_match_contigs_to_probes.py script.

Off-targets reads may contain sequences from loci traditionally used in phylogenetic studies (herein referred to as ‘legacy loci’) (e.g. [32,49–51]). As such, we also extracted 15 mitochondrial and two nuclear legacy loci from off-target contigs in our target capture data for comparison between capture protocols (see electronic supplementary material, appendix S1 for further details on background, methods and results pertaining to legacy loci).

As one part of the experiment, we also wanted to quantify the effect of different tiling strategies (approximately 1.33x versus approximately 2x tiling density) on locus recovery. As most loci with baits tiled differently also exhibited major differences in bait-target divergences (see Results), we were not able to directly measure the effect of tiling strategy for most loci. However, some loci (i.e. 40 out of 2566) captured with Pentatomomorpha-derived baits had low average minimum bait-target divergences similar to loci captured with Coreoidea-derived bait (see Results). Thus, we had a limited opportunity to explore the effect of tiling strategy on read depth while controlling for bait-target divergences. For this, we selected loci captured with Coreoidea- or Pentatomomorpha-derived baits that had divergences ranging from 0.05 to 0.10 and calculated read depth.

Sequencing depth between different sequence lanes/runs may affect how many loci are recovered or the proportion of on-target reads. Furthermore, the effective sample size across different sequencing efforts can vary as some samples may fail or have poor sequencing outcomes, which can have an impact on sequencing depth across samples. Our samples were subjected to three different sequencing efforts, with each producing different effective sample sizes: (i) 99 samples combined for previously sequenced samples subjected to the standard capture conditions, (ii) 96 samples combined for previously sequenced samples subjected to the TD-60 capture conditions, and (iii) 88 samples combined for sequenced samples subjected to the different target capture conditions conducted in this study. To investigate the influence of different sequencing depths on our metrics of capture success, we equalized sequencing depth by subsampling 2 000 000 raw reads generated under the different capture protocols for 24 taxa that were preserved fresh using Seqtk v. 1.3 (https://github.com/lh3/seqtk) (random seed [-s option] = 100). Eleven taxa were not included in this analysis because at least one of the target capture protocols for each of these taxa was associated with fewer than 3 000 000 raw reads total; this drastically lowered the sample size of available samples that were preserved dried (four taxa remaining), and thus, we excluded dried samples. The subsampled reads were then processed and evaluated as described above to determine if patterns observed in the subsampled dataset differed from what was observed in the original data.

### 2.5. Statistical analyses

Statistical analyses using linear mixed models (LMMs), generalized linear models (GLMs) and generalized linear mixed models (GLMMs) were performed using *lme4* v. 1.1.30 [52] in R v. 4.1.2 [53]. We excluded dried samples from statistical analyses due to very low sample size for some target capture protocols, with the exception that they were included with fresh samples when analysing bait-target divergences, since this was independent of target capture protocol. We refrained from treating library quality as a factor in analyses, since this was used as an *a posteriori* criterion for selecting samples to be included in a second capture protocol. We also did not include the different sequencing efforts across samples as a random effect since it has been suggested that random effect terms should have at least five levels for inclusion [54] (but see [55]). However, to account for potential effects of different sequencing efforts, we subsampled our data as described in the previous section to normalize sequencing depth across sequencing efforts. For LMM and GLMM analyses, we treated target capture protocol as a fixed effect. Because the same sample was subjected to two capture protocols, we included replication as a random effect. The GLM analyses treated bait design strategy as a fixed effect. For all analyses, we performed simultaneous tests for general linear hypotheses using Tukey contrasts for multiple comparisons of means using the *multcomp* v. 1.4.20 R package [56]. See Data accessibility for scripts used for statistical analyses.

#### 2.5.1. Specific details of the statistical analyses for the entire dataset

We conducted LMM analyses on read depth across all targeted loci regardless of bait design strategy, as well as when data were partitioned by bait design strategy. The response variable was log-transformed to achieve a normal distribution.

The response variables for GLM analyses were the average minimum bait-target divergences and the average read depth per locus across loci with low bait-target divergences (i.e. tiling strategy), with the latter log-transformed to achieve a more normal distribution. We used a binomial error structure with the average minimum bait-target divergences, while a Gaussian structure was used for the analysis investigating bait tiling strategy.

For GLMM analyses, we used a Poisson error structure with the following response variables: median lengths of loci targeted by Pentatomomorpha-derived baits and number of putative paralogues of loci targeted by transcript- and Pentatomomorpha-derived baits, as well as by PC dual baits. Due to evidence of overdispersion when using a Poisson error structure, we used the negative binomial family for the following response variables: total number of raw reads; total number of contigs; median contig length; median lengths of loci targeted by exon-, transcript- and Coreoidea-derived baits, as well as PC dual baits; and number of putative paralogues of loci targeted by exon- and Coreoidea-derived baits. A binomial family was used for analyses involving the proportion of on-target reads (with an observation-level random effect due to evidence of overdispersed data [57]), proportion of target loci recovered (with an observation-level random effect for those targeted by exon-, Coreoidea- and Pentatomomorpha-derived baits) and proportion of legacy loci recovered.

#### 2.5.2. Specific details of the statistical analyses for the subsampled dataset

For our dataset that subsampled reads, we performed statistical analyses similar to those described above for the entire dataset. For LMM analyses, only read depth data from the Pentatomomorpha-derived bait treatment were log-transformed to achieve a normal distribution (other treatments were normally distributed).

For GLMM analyses, many of our response variables were analysed similarly as in the previous section. Here, we only highlight cases where four GLMMs were constructed slightly differently. First, we did not include an observation-level random effect for GLMMs with the proportion of target loci recovered by exon- and Coreoidea-derived baits, as there was no evidence of overdispersed data. Second—and for similar reasoning—we used a Poisson error structure with the number of putative paralogues of loci targeted by exon- and Coreoidea-derived baits.

## 3. Results

### 3.1. Greater raw read and assembled contig yield at low hybridization temperatures

**Figure 3.**
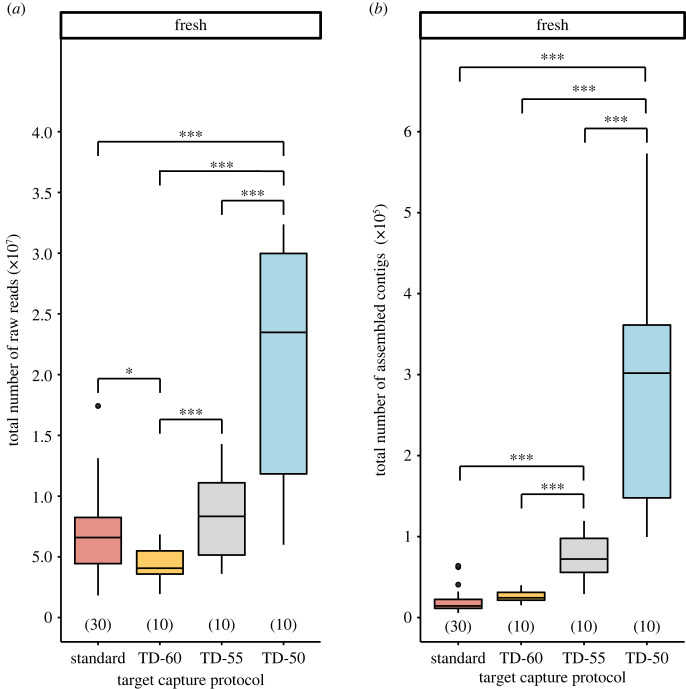
Effects of target capture protocols on the (*a*) total number of raw reads and (*b*) assembled contigs for samples preserved fresh. Numbers in parentheses above *x*-axis denote sample size. Single and triple asterisks denote statistically significant pairwise comparisons, with *p* < 0.050 and *p* < 0.001, respectively.

Overall, lower hybridization temperatures generated significantly more raw sequence reads for samples preserved fresh (electronic supplementary material, table S3; figure 3*a*), with dried samples exhibiting similar trends (electronic supplementary material, figure S1*a*). Of the 39 samples in our dataset, 26 had more raw reads sequenced when these samples were subjected to lower hybridization temperatures in pairwise comparisons (median increase = 272%) (electronic supplementary material, table S3). The TD-50 capture protocol produced the most raw reads for freshly preserved samples (figure 3*a*), while the TD-50 and TD-55 produced the most for dried samples (electronic supplementary material, figure S1*a*). Three degraded (i.e. preserved dried) samples failed or nearly failed to produce any raw reads under the standard protocol, but they had greater than 33 000 reads sequenced at a modestly lower hybridization temperature (TD-60) (electronic supplementary material, table S3).

In general, touchdown protocols also produced a greater total of assembled contigs and nucleotides (electronic supplementary material, table S3 and figure S1*b*; figure 3*b*). Contigs had similar ranges of median lengths between capture protocols, but, for freshly preserved samples, all the touchdown protocols had significantly longer median contig lengths than the standard protocol while there were no statistical differences between touchdown protocols (electronic supplementary material, figure S1*c*). For dried samples, median contig length exhibited increasing trends as hybridization temperature was lowered (electronic supplementary material, figure S1*c*). Data generated under protocols with lower hybridization temperatures yielded some of the longest contigs recovered (electronic supplementary material, table S3). Thus, while lower hybridization temperatures varied in their success when looking at a given taxon, the lowest hybridization temperature resulted in the most raw reads and contigs across samples, on average.

### 3.2. Bait-target distances differ between bait design strategies

We found that loci targeted by either Coreoidea exon- and transcript-derived bait sequences followed similar trends in our experiment, as well as shared similar average minimum bait-target divergences (electronic supplementary material, figure S1*d*). Due to this, as well as shared genomic resources during bait design and the relatively few number of loci, we combined results from the exon- and transcript-targeted loci together, which we refer to as Coreoidea-derived baits (for separate exon- and transcript-targeted locus results, see electronic supplementary material). Similar average minimum bait-target divergences were observed for loci captured by Coreoidea-derived baits and PC dual baits, and these were significantly lower and exhibited less variation compared with those captured by Pentatomomorpha-derived baits (electronic supplementary material, figure S1*d*).

### 3.3. Proportion of reads on-target and read depth decrease at low hybridization temperatures

**Figure 4.**
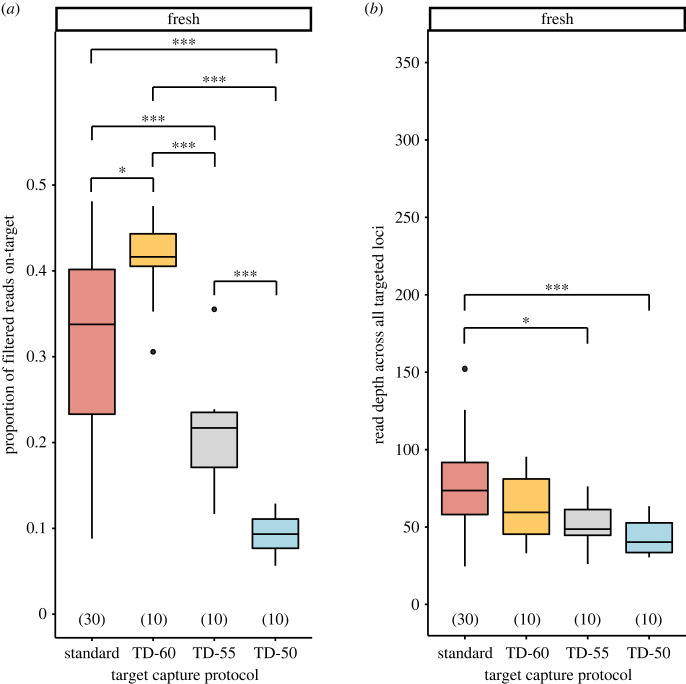
Effects of target capture protocols on (*a*) the proportion of filtered reads on-target and (*b*) read depth across all targeted loci for samples preserved fresh. Numbers in parentheses above *x*-axis denote sample size. Single and triple asterisks denote statistically significant pairwise comparisons, with *p* < 0.050 and *p* < 0.001, respectively. Abbreviations: PC dual baits, loci targeted by both Pentatomomorpha- and Coreoidea-derived baits; figure 1.

There was a general increase in the number of filtered on-target reads in pairwise comparisons as hybridization temperature was lowered (electronic supplementary material, table S4). Despite this, the TD-50 capture protocol had a significantly lower proportion of on-target reads across all targeted loci for freshly preserved samples (figure 4*a*). When partitioning by bait design strategy, this pattern remained for freshly preserved samples, but with the TD-55 protocol also resulting in a lower proportion of on-target reads compared with the TD-60 and standard protocols (electronic supplementary material, figure S2). Dried samples did not show any noticeable trends as hybridization temperature was lowered, with the exception that all touchdown capture protocols had a higher proportion of on-target reads compared with the standard protocol (electronic supplementary material, figure S2).

In pairwise comparisons, there was frequently a decrease in read depth at lower temperatures regardless of bait design strategy, with overall read depth significantly different between the TD-50 and TD-55 protocols and the standard protocol for freshly preserved samples only (electronic supplementary material, table S4; figure 4*b*). However, this pattern was not consistently observed when partitioning by bait design strategy (electronic supplementary material, figure S3): (i) the TD-55 capture protocol did not have a significantly lower read depth for loci targeted by Coreoidea-derived baits compared with the standard protocol; (ii) read depth of loci targeted by PC dual baits was not significantly different across capture protocols; and (iii) Pentatomomorpha-derived baits from the TD-60 and TD-55 protocols were significantly lower than the standard, but were not significantly different from each other nor the TD-50 protocol. For dried samples, read depth did not appear to exhibit any trends across capture protocols, with the exception that lower temperatures exhibited greater read depth for loci targeted by Coreoidea-derived and PC dual baits (electronic supplementary material, table S4 and figure S3).

### 3.4. Low hybridization temperatures improve locus recovery and lengths

**Figure 5.**
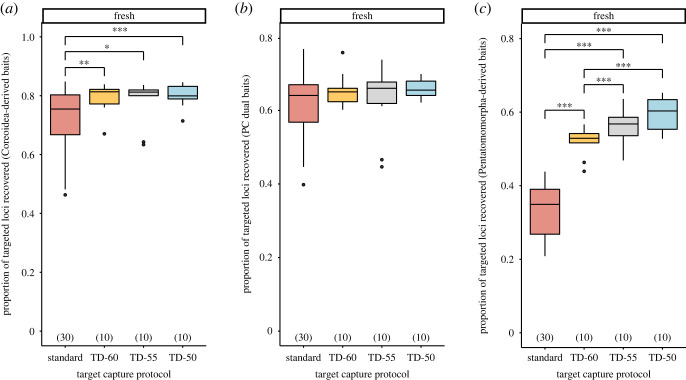
Effects of target capture protocols on the proportion of recovered loci targeted by (*a*) Coreoidea-derived baits, (*b*) PC dual baits and (*c*) Pentatomomorpha-derived baits for samples preserved fresh. Numbers in parentheses above *x*-axis denote sample size. Single, double and triple asterisks denote statistically significant pairwise comparisons, with *p* < 0.050, *p* < 0.010 and *p* < 0.001, respectively. Abbreviations: see figures 1 and 4.

**Figure 6.**
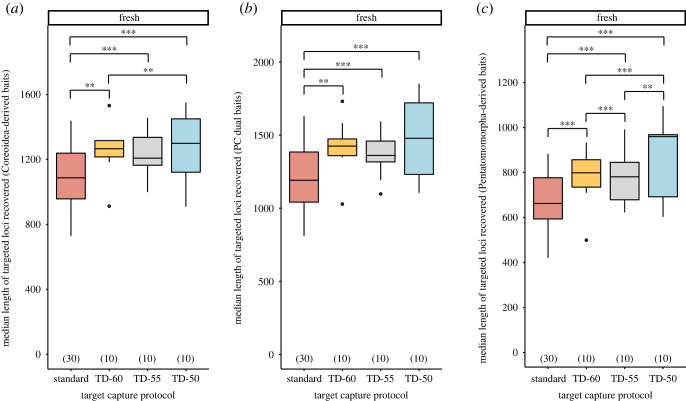
Effects of target capture protocols on the median length of recovered loci targeted by (*a*) Coreoidea-derived baits, (*b*) PC dual baits and (*c*) Pentatomomorpha-derived baits for samples preserved fresh. Numbers in parentheses above *x*-axis denote sample size. Single, double and triple asterisks denote statistically significant pairwise comparisons, with *p* < 0.050, *p* < 0.010 and *p* < 0.001, respectively. Abbreviations: see figures 1 and 4.

Lower hybridization temperatures produced significantly more loci targeted by Coreoidea- and Pentatomomorpha-derived baits compared with the standard temperature for freshly preserved samples, with the TD-50 protocol resulting in the highest proportion of loci recovered (electronic supplementary material, tables S5–S8; figure 5*a–c*). The median lengths of targeted loci were significantly longer when the TD-50 protocol was used compared with higher hybridization temperatures for freshly preserved samples, regardless of bait design strategy (electronic supplementary material, tables S5–S8; figure 6*a*–*c*). Dried specimens did not exhibit any trends with respect to target capture protocol and the proportion of targeted loci recovered or the median lengths of targeted loci (electronic supplementary material, tables S5–S8 and figures S4 and S5).

### 3.5. Recovery of putative paralogues at low hybridization temperatures depends on bait design strategy

**Figure 7.**
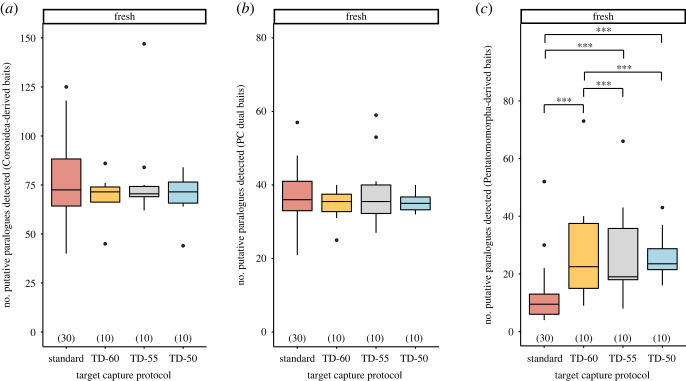
Effects of target capture protocols on the number of putative paralogues of loci captured by (*a*) Coreoidea-derived baits, (*b*) PC dual baits and (*c*) Pentatomomorpha-derived baits for samples preserved fresh. Numbers in parentheses above *x*-axis denote sample size. Triple asterisks denote statistically significant pairwise comparisons, with *p* < 0.001. Abbreviations: see figures 1 and 4.

We did not observe any patterns with respect to hybridization temperatures and the number of putative paralogues detected for Coreoidea-derived and PC dual baited strategies for samples preserved fresh (electronic supplementary material, tables S5–S7; figure 7*a,b*). By contrast, we observed increases in putative paralogues associated with loci targeted by the Pentatomomorpha-derived baits as hybridization temperatures decreased (electronic supplementary material, table S8; figure 7*c*). We did not observe any noticeable trends with respect to hybridization temperatures and putative paralogues detected by any bait strategy for dried samples (electronic supplementary material, tables S5–S8 and figure S6).

### 3.6. Additional measures and parameters investigated

#### 3.6.1. Greater bait tiling improves read depth

We quantified the effect of different tiling strategies on one aspect of locus recovery, read depth. Most loci with baits tiled differently exhibited major differences in bait-target divergences (electronic supplementary material, figure S1*d*), but 40 loci captured with Pentatomomorpha-derived baits had low average minimum bait-target divergences similar to loci captured with Coreoidea-derived bait. We selected loci captured with Coreoidea- or Pentatomomorpha-derived baits that had divergences ranging from 0.05 to 0.10 to control for bait-target divergences and explore the effect of tiling strategy on read depth. We found that loci captured by Coreoidea-derived baits (approximately 2x tiling) had significantly greater average read depth than those captured by Pentatomomorpha-derived bait (1.33x tiling; electronic supplementary material, figure S7).

#### 3.6.2. Minor impacts of sequencing depth on freshly preserved samples

To investigate the influence of sequencing depth on our metrics of capture success, we evaluated whether the capture success metrics for 24 freshly preserved taxa were still congruent when reads were subsampled evenly (electronic supplementary material, tables S9–S14 and figures S8–S13). In general, patterns across hybridization temperatures remained congruent for most metrics compared with when reads were not subsampled, including: total number of assembled contigs, proportion of filtered reads on-target, proportion of targeted loci recovered and number of putative paralogues found (electronic supplementary material, tables S9–S14 and figures S8*a*, S8*c*, S9, S10 and S13).

By contrast, the median length of assembled contigs was not significantly different across hybridization temperatures but exhibited similar ranges of variation and general trends compared with those when reads were not subsampled (electronic supplementary material, table S9 and figure S8*b*). We also found read depth across targeted loci to decrease significantly as hybridization temperatures were reduced, regardless of bait design strategy (compared with little or no significant reductions when reads were not subsampled) (electronic supplementary material, tables S9 and S10 and figures S8*d* and S10). Lastly, the median lengths of targeted loci recovered were significantly shorter at lower hybridization temperatures (electronic supplementary material, tables S11–S14 and figure S12), in contrast with the significantly longer loci at these same temperatures when reads were not subsampled.

## 4. Discussion

Invertebrate target capture studies employing fixed, high hybridization temperatures often recover relatively low proportions of targeted loci. For many taxonomic groups, like the leaf-footed bugs studied here, genomic resources are lacking or too limited to adequately optimize target capture baits to improve locus recovery. Furthermore, optimizing or redesigning target capture baits to improve locus recovery may result in more bait sets that are too narrowly targeted from a taxonomic perspective (e.g. a family) when there can be advantages of making bait sets more broadly applicable (e.g. a superfamily, infraorder or higher ranks). Thus, modifying target capture conditions may be an effective approach to improve the success of capture experiments across a broader taxon sampling.

Our evaluation of four different in-solution capture protocols found hybridization temperatures lower than the standard 65°C led to more assembled contigs and improved recovery of targeted loci, which held true even after normalizing sequencing depth. While this improvement was observed for loci targeted by the less divergent Coreoidea-derived baits, this was even more apparent for loci targeted by the more divergent Pentatomomorpha-derived baits. Such an improvement occurred despite touchdown capture ending at 50°C generally having a negative impact on the proportion of on-target reads and read depth, as well as increased numbers of putative paralogous loci when using the Pentatomomorpha-derived baits. We also found that lower hybridization temperatures also led to a greater recovery of other common loci of historical use from off-target reads (particularly mitochondrial regions), which is an alternative approach to designing custom baits for such loci or relying on low-coverage genome sequencing (see electronic supplementary material, appendix S1 for more information on this topic and specific results). Future studies with invertebrates should consider optimizing *in vitro* target capture conditions to accommodate hybridization temperatures as low as 50°C as a cost-effective solution to improve the successful recovery of targeted loci (albeit with some costs), while also providing opportunities to gain additional data.

### 4.1. Benefits and considerations for using low hybridization temperatures and greater bait tiling

Previous studies suggest that the efficacy of target capture decreases when bait-target divergences exceed 5–10% [2,24,58], although successful capture has been reported when divergences are much higher (e.g. 40% in [5]). The Pentatomomorpha-derived baits used here were derived from two taxa in the same infraorder as the leaf-footed bugs [33], but these taxa diverged from coreoids at approximately 160–230 Ma [36–38]. The Pentatomomorpha-derived baits generally exhibited high levels of divergence from the targeted loci in coreoids, sometimes as high as that seen in [5]. By reducing target capture stringency via lower hybridization temperatures, a greater mismatch tolerance between divergent baits and targets can lead to improved locus recovery, which our study supports. Given that our Coreoidea-derived baits or PC dual baits were designed from coreoid transcriptomes, we were able to target loci with much lower divergences from their respective baits (less than 10%).

Tissue quality appears to have some influence on the trends we observed with respect to hybridization temperatures and many capture success metrics. Our three touchdown hybridization temperatures improved locus recovery for degraded, dried samples when compared with the fixed standard 65°C, but recovery did not appear to differ when comparing across the touchdown protocols. For samples with highly fragmented DNA, it may be that the hybridization temperature of baits to bind to short target DNA (shorter than DNA usually targeted from fresh samples) only requires a modest reduction to obtain the maximum amount of targeted data possible. Furthermore, slightly lowering hybridization temperature may have the benefit of recovering enough loci to ensure a taxon can be represented in a final analysis where missing data is minimized. Thus, based on our assessment, employing capture approaches with hybridization temperatures less than 65°C generally has positive outcomes, especially with samples of low quality.

Bait tiling strategy also appears to have played a role, although we could not thoroughly explore its effects on many of our metrics. Despite some loci targeted by the Pentatomomorpha-derived baits exhibiting similar levels of divergences as those targeted by Coreoidea-derived baits, we found that a modest increase in bait tiling density (i.e. the latter set of baits) was associated with greater read depth of the targeted loci. Lower bait-target divergences and/or greater tiling of baits across targeted loci probably explain the relatively high proportion of loci recovered by these Coreoidea-derived baits compared with those recovered with Pentatomomorpha-derived baits.

If locus recovery is the main goal of invertebrate capture studies (especially if bait-target divergences exhibit a broad range of variation as in our study), using less stringent hybridization temperatures can be particularly beneficial when baits and targets are expected to exhibit high divergences and/or when baits are tiled at a low density. While the lowest hybridization temperature tested (TD-50) resulted in the best target locus recovery—especially for the more divergent target loci—this result is probably due in part to the higher amount of data (i.e. reads and contigs) obtained for this treatment. However, we found that the TD-60 protocol provided the best balance with respect to the proportion of on-target reads and lower hybridization stringency allowing for maximal target locus recovery when we subsampled raw reads across all capture conditions to normalize sequencing depth. Thus, in future studies attempting to maximize target data for many samples with relatively lower sequencing depth, the TD-60 protocol may be preferable, but if sequencing depth is not a critically limiting factor, the TD-50 protocol may be a more desirable option.

### 4.2. Could hybridization temperatures be further reduced?

Further studies are needed to determine if hybridization temperatures lower than 50°C could be beneficial for locus recovery in target capture studies. We expect it is likely that the benefits of even lower temperatures will eventually be outweighed by the costs. Lowering hybridization temperature to reduce target capture stringency is expected to result in a greater proportion of off-target than on-target sequences in capture data due to greater mismatch tolerance. Previous studies have observed this pattern ([23,24], ancient and archival specimens), which we also support here. More off-target data may be considered undesirable for many studies, and for phylogenomic studies, this could include potential sequences deemed paralogous to target data. Our results demonstrate that relaxing the specificity between bait and targets increases the recovery and detection of putative paralogous loci at low hybridization temperatures. We expect this pattern of increasing recovery of paralogous loci to become even greater at temperatures lower than 50°C with little or no improvement in target locus recovery. We found more potential paralogues recovered for loci targeted by Pentatomomorpha-derived baits, but not other targeted loci, which may be related to the degree of bait-target divergences or tiling strategy. Thus, for capture studies using baits expected to be quite divergent from ingroup taxa, lower hybridization temperatures can result in more targets recovered, but possibly at the cost of recovering more paralogous loci and a loss of some corresponding true targets.

Accurate paralogue detection steps are very important regardless of hybridization temperatures (and how many paralogues are expected to be recovered) as any undetected paralogues have the potential to mislead phylogenetic inference. When putative paralogues are detected in some sequence processing pipelines like PHYLUCE, both the target and putative paralogue are excluded from further analysis, so even targeted data may be excluded from final analyses. Hybridization temperatures lower than 50°C could exacerbate this problem due to greater recovery of putative paralogues. However, further evaluation of putative paralogues and potential targets can often prevent the loss of data, especially since bioinformatic pipelines like PHYLUCE can report information on multiple contigs matching to a bait. This then allows analysing sequences more carefully with bioinformatic tools like BLAST, inspecting alignments containing reference sequences or refining orthologue binning procedures in bioinformatic pipelines to also use untargeted paralogues in analyses. Thus, the inclusion of paralogues may not be a problem (and could add additional loci for analyses), as long as these are accurately identified and addressed.

### 4.3. Capture duration probably not a confounding factor

Our touchdown capture protocols were performed with longer durations than the standard protocol (36 versus 24 h, respectively), and one of these touchdown protocols had four incremental temperature decreases every 9 h instead of three incremental decreases every 12 h (figure 1). Thus, the total capture duration and duration of the incremental temperature decreases might be considered confounding factors in our experiment. However, two of our target capture protocols (TD-60 and TD-55) used the same timing (36-hour total duration, with temperature decreases every 12 h), and, as with all other capture protocols we used, the lower hybridization temperature (i.e. TD-55) improved target capture success for some metrics (such as number of raw reads, contigs and number of recovered loci targeted by Pentatomomorpha-derived baits) while exhibiting negative effects on other metrics (e.g. less reads on-target or more putative paralogues of recovered loci targeted by Pentatomomorpha-derived baits). Thus, the duration of target capture or the duration of bait-target hybridization at specific temperatures does not appear to be a major confounding factor in our study.

### 4.4. Considerations for using transcript-derived baits

We employed a bait design procedure in which coreoid transcriptomes were used to identify individual exons that baits would target, as well as entire transcripts that could contain multiple exons with baits potentially dissected by introns (i.e. a single bait that is derived from two exons). Based on our metrics—irrespective of target capture conditions (particularly the number of targeted loci recovered)—we found that our exon-derived baits were highly successful (78% recovery, on average) compared with the transcript-derived baits (33% recovery, on average). This finding indicates that most of the 58 loci targeted by transcript-derived baits could not be captured. The poor *in vitro* performance of our transcript-derived baits may be due to transcript sequences comprising many short exons leading to many baits dissected by introns. This could result in multiple assembled contigs if the entire intron(s) was not sequenced, which would result in the associated transcript baits matching multiple ‘different’ contigs in PHYLUCE and exclusion of these sequences due to putative paralogy. Thus, our transcript-derived baits do not appear very useful in Coreoidea target capture studies, and our transcript bait design strategy may not be suitable for other groups in general unless exon-intron boundaries are known or different bioinformatic strategies are used.

## 5. Conclusion

Our study primarily focused on the effects of a single target capture parameter, i.e. hybridization temperature, on several metrics of capture success in an invertebrate protein-coding dataset. However, given that our baits were derived from different genomic resources, we were also able to explore the role of bait-target divergence and bait tiling strategy in our experiment, as well as tissue quality. While hybridization temperature, bait-target divergence and bait tiling strategy had effects on some of our metrics, we recognize other parameters probably affect capture efficacy and remain to be thoroughly investigated in target capture experiments similar to ours (i.e. in-solution capture, sample quality, etc.). Such parameters may also include base genomes used in probe design [18], GC content of baits [22,59], amount of baits used [23] and post-hybridization washing stringency [5], among many others. Regardless, the following, widely applicable principals can be derived from our study: (i) low hybridization temperature improves target recovery in invertebrate material—especially those with high bait-target divergences—without major negative impacts overall; (ii) low hybridization temperature can also retrieve other potentially useful data for comparative analyses; (iii) low hybridization temperature offers particular benefits when sequencing low quality or degraded material; and (iv) tiling depth has a substantial impact on read depth. We hope future practitioners of target capture methods employ these simple *in vitro* modifications to improve recovery of loci and generate higher quality, more comprehensive phylogenies.

## Data Availability

Newly generated sequence read files are available on NCBI's Sequence Read Archive (SRA) under BioProject PRJNA763985. For previously published sequence read data, see NCBI SRA BioProjects PRJNA531965 [34], PRJNA546248 [30] and PRJNA609116 [29]. Pentatomomorphan- and Coreoidea-derived baits, as well as scripts used for statistical analyses, are accessible on FigShare under the project titled ‘Coreoidea UCE bait set’: https://figshare.com/projects/Coreoidea_UCE_bait_set/161416. The data are provided in the electronic supplementary material [60].
